# Concepts of order: Why is ordinality processed slower and less accurately for non-consecutive sequences?

**DOI:** 10.1177/17470218231220912

**Published:** 2024-01-11

**Authors:** Declan Devlin, Korbinian Moeller, Iro Xenidou-Dervou, Bert Reynvoet, Francesco Sella

**Affiliations:** 1 Loughborough University, Loughborough, UK; 2 Leibniz-Institut für Wissensmedien, Tübingen, Germany; 3 LEAD Graduate School & Research Network, Tübingen, Germany; 4 KU Leuven, Leuven, Belgium

**Keywords:** Number order processing, numerical cognition, order judgement, reverse distance effect, ordinal knowledge

## Abstract

Both adults and children are slower at judging the ordinality of non-consecutive sequences (e.g., 1-3-5) than consecutive sequences (e.g., 1-2-3). It has been suggested that the processing of non-consecutive sequences is slower because it conflicts with the intuition that only count-list sequences are correctly ordered. An alternative explanation, however, may be that people simply find it difficult to switch between consecutive and non-consecutive concepts of order during order judgement tasks. Therefore, in adult participants, we tested whether presenting consecutive and non-consecutive sequences separately would eliminate this switching demand and thus improve performance. In contrast with this prediction, however, we observed similar patterns of response times independent of whether sequences were presented separately or together (Experiment 1). Furthermore, this pattern of results remained even when we doubled the number of trials and made participants explicitly aware when consecutive and non-consecutive sequences were presented separately (Experiment 2). Overall, these results suggest slower response times for non-consecutive sequences do not result from a cognitive demand of switching between consecutive and non-consecutive concepts of order, at least not in adults.

## Introduction

To work confidently with symbolic numbers, one must be able to comprehend both cardinality (i.e., overall magnitude) and ordinality (i.e., relative position within an ordered sequence). For example, it is necessary to understand both that six is greater than four in terms of magnitude, but also that five comes after four and before six in terms of ordinality. Although cardinality has typically received more research attention than ordinality, order processing has become an increasingly important topic within numerical cognition research (for a review, see Lyons et al., 2016).

In particular, order processing has been frequently considered as a predictor of numerical skills such as arithmetic (Malone et al., 2021; Orrantia et al., 2019). In fact, by second grade, order processing has been shown to become a stronger predictor of arithmetic performance than magnitude processing (Lyons et al., 2014; Sasanguie & Vos, 2018). Moreover, deficits in order processing have been frequently associated with numerical learning difficulties such as developmental dyscalculia (Decarli et al., 2023; Morsanyi et al., 2018). However, despite its apparent relevance, the cognitive mechanisms underlying order processing and their exact contributions to numerical development remain debated (Devlin et al., 2022).

Currently, the primary method of investigating the mechanisms underlying order processing involves using order judgement tasks in which participants have to classify sequences of digits (e.g., 1-2-3) as either correctly or incorrectly ordered (Dubinkina et al., 2023; Finke et al., 2021; Muñez et al., 2022; Wong et al., 2022). A noteworthy feature of these tasks is that they often produce a reverse distance effect whereby consecutive sequences (e.g., 1-2-3) are processed faster than non-consecutive sequences (e.g., 1-3-5; see Goffin & Ansari, 2016; Vogel et al., 2017, 2021). This effect is reversed relative to the standard distance effect observed in magnitude comparison tasks whereby judgements become faster as the distance between digits increases (Moyer & Landauer, 1967); although, viewing the reverse distance effect in order processing as analogous to the standard effect in magnitude processing has been discouraged (see Devlin et al., 2022). A common explanation for the reverse distance effect is that it likely results from the processing of consecutive sequences being facilitated by memory retrieval as consecutive sequences are more familiar and thus more easily retrieved from memory (Vos et al., 2017, 2021). Consequently, when interpreted as a facilitation effect, the reverse distance effect suggests that memory-retrieval processes likely mediate the association between order processing and arithmetic performance (Dubinkina et al., 2021; Sasanguie & Vos, 2018; Sommerauer et al., 2020).

In recent years, however, alternative explanations of the reverse distance effect have been considered. One proposed alternative to the facilitation account stems from suggestions that young children may initially believe that “in order” refers only to sequences that directly match the count-list (Gilmore & Batchelor, 2021; Hutchison et al., 2022). Therefore, it has been argued that the reverse distance effect might actually result from the processing of non-consecutive sequences being impeded due to conflicting with this intuition, rather than from consecutive sequences being facilitated by memory retrieval (Gattas et al., 2021). However, although both the facilitation and count-list interpretations of the reverse distance effect seem plausible, there has been limited direct comparison between them (Devlin et al., 2022).

Although the count-list interpretation of the reverse distance effect has received less research attention than the facilitation account, recent findings have supported this perspective. For instance, children between the ages of 4 and 6 years were reportedly less likely to endorse the use of ordinal language (i.e., “before” or “after”) for non-consecutive numbers (Hurst et al., 2022). In fact, during order judgement tasks, many children between the ages of 6 and 9 years (mean age = 7.58 years) appear to consistently classify non-consecutive sequences as not in order (Gilmore & Batchelor, 2021). Furthermore, Hutchison et al. (2022) observed that children’s transition from consistently rejecting to consistently verifying non-consecutive sequences occurred as a sudden qualitative shift, rather than through incremental improvement; suggesting that expanding one’s concept of order to include non-count-list sequences presents a developmental challenge that children need to overcome. Therefore, it is plausible that our early intuitions about order could have a lasting impact on how we process and conceptualise ordinality, which may continue into adulthood.

To test whether people are predisposed to consider non-consecutive sequences as not in order, Gattas et al. (2021) varied the instructions given to participants taking an order judgement task. Surprisingly, they found that adult participants were faster at classifying non-consecutive sequences as not in order than they were at classifying them as in order. Therefore, not only does this finding support the presence of an intuition that “in order” refers only to sequences that directly match the count-list, but it also suggests the reverse distance effect likely results from having to suppress this intuition, rather than from consecutive sequences being facilitated by memory retrieval (Gattas et al., 2021).

Speculating on the exact process by which early intuitions about ordinality could slow response times for non-consecutive sequences, Gattas et al. (2021) proposed that adults may implement a two-step process during order processing tasks whereby they first check whether a sequence directly matches the count-list. If so, the sequence can be instantly verified, otherwise it must undergo a second round of checks. This second round may involve, for example, implementing strategies such as sequential magnitude comparisons to determine whether the sequence is correctly ordered. Consequently, from a count-list perspective, this need for non-consecutive sequences to undergo additional checks may account for why non-consecutive sequences are processed slower than consecutive sequences (Gattas et al., 2021).

A key implication of the count-list interpretation of the reverse distance effect is that it suggests order processing performance may provide insight into how an individual conceptualises ordinality. For example, those who display a strong reverse distance effect may possess a rigid conceptualisation of ordinality limited primarily to ascending consecutive sequences. In contrast, those who display no reverse distance effect may have a very flexible concept of ordinality. Therefore, the count-list perspective arguably suggests a much more fundamental role of ordinal understanding in numerical development than simply the shared use of memory-retrieval strategies implied by the facilitation account. Accordingly, Hutchison et al. (2022) argued that the order processing deficits observed in dyscalculia may result from a specific difficulty in expanding one’s concept of order beyond the count-list. Consistent with this, they noted that people with dyscalculia appear to show specific difficulty with judging the ordinality of non-consecutive sequences (Morsanyi et al., 2018). This interpretation seems compelling because it parallels the need to overcome similar counterproductive intuitions in other aspects of early mathematical development (Gattas et al., 2021). For example, we also need to overcome our intuitions based on positive integers when working with negative numbers and fractions (Ni & Zhou, 2005; Siegler & Lortie-Forgues, 2014).

However, although the count-list perspective provides a compelling explanation for the reverse distance effect, recent studies have also challenged this interpretation. For instance, Devlin et al. (2022) proposed an alternative explanation for why adults appeared faster at classifying non-consecutives sequences as not in order. More specifically, they argued that when classifying non-consecutive sequences (e.g., 2-4-6) as correctly ordered, all three digits must be processed to verify this. In contrast, when classifying them as incorrectly ordered, only the first two digits need to be processed as these are necessarily non-consecutive. Similarly, when only considering consecutive sequences as ordered, all non-ordered sequences could also be rejected after the first two digits because these two digits were always either non-consecutive (e.g., 4-6-5) or descending (e.g., 4-3-5). Consistent with this, Gattas et al. (2021) observed faster response times for both non-consecutive and non-ordered sequences. Whereas, if this faster processing resulted from matching participants’ intuitions about order, any such improvement in response times would arguably only be expected for non-consecutive sequences, rather than for both non-consecutive and non-ordered sequences (Devlin et al., 2022).

A second issue highlighted by Devlin et al. (2022) concerns the relationship between mathematics performance and the presence of the reverse distance effect. Recent findings have suggested that not everyone necessarily displays a reverse distance effect during order processing (Sasanguie & Vos, 2018; Vogel et al., 2021). Therefore, in this context, it might be argued that if the reverse distance effect reflects a person’s difficulty in overcoming their early intuitions about order, those who show this effect would be expected to perform worse in mathematics. This is because they would also be expected to struggle with overcoming similar early established counterproductive intuitions such as the natural number bias (Ni & Zhou, 2005). However, Vogel et al. (2021) found that the relationship between order processing and mathematics was actually stronger in those who displayed a reverse distance effect compared to those who did not. Therefore, it is unclear how the count-list perspective can account for this finding (Devlin et al., 2022).

Despite these limitations, however, there remains strong evidence that many children consider non-consecutive sequences as not in order (Gilmore & Batchelor, 2021; Hutchison et al., 2022). Moreover, because this count-list perspective suggests a much more fundamental role of ordinal understanding in numerical development than the facilitation perspective, it certainly warrants further consideration (Devlin et al., 2022). In this context, a possible alternative explanation is that these children may understand conceptually that non-consecutive sequences are correctly ordered, but simply struggle to indicate this due to the cognitive demands of the task. For instance, the order judgement task requires not only understanding that both consecutive and non-consecutive are valid concepts of order, but also requires repeatedly switching between these two concepts. Therefore, it seems plausible this switching process may increase the cognitive demands of the task. Accordingly, the task may be too demanding for a subset of children, thus accounting for why they consistently failed to verify non-consecutive sequences as ordered. Moreover, if the tendency to reject non-consecutive sequences does result from the task being too cognitively demanding, children who struggle on this task should still be able to indicate non-consecutive sequences as ordered in other contexts. Supporting this, Bugden and Brannon (2021) found that children who consistently rejected non-consecutive sequences during a standard order judgement task were still able to correctly produce ordered non-consecutive sequences during an order production task (for other examples of order production tasks, see Xu et al., 2023; Xu & LeFevre, 2021).

A key prediction from this cognitive demand perspective is that reducing the hypothesised additional demand of the task should improve order processing performance, particularly on non-consecutive sequences. Accordingly, if the cognitive demand stems from switching between consecutive and non-consecutive concepts of order, then presenting consecutive and non-consecutive sequences separately should eliminate this demand. Alternatively, from a two-step perspective, presenting these sequence types separately should eliminate the need for adults to implement a two-step process involving first checking whether a sequence matches the count-list. We tested these hypotheses across two experiments. In the first experiment, we had adult participants complete an order judgement task under two conditions: one in which consecutive and non-consecutive sequences were presented within the same block (mixed condition), and another in which they were presented in separate blocks (blocked condition). We expected that response times would be faster in the blocked condition compared with the mixed condition. In particular, we predicted this improvement in the blocked condition would be greater for non-consecutive sequences than for consecutive sequences.

## Experiment 1

### Method

#### Participants

A total of 100 UK-based participants (*M*_age_ = 39.06 years, *SD* = 14.35) were recruited via Prolific.co. Each participant received £1.67 for participating. Both experiments in this study received ethical approval from Loughborough University’s ethics committee.

#### Order judgement task

**Figure 1.**
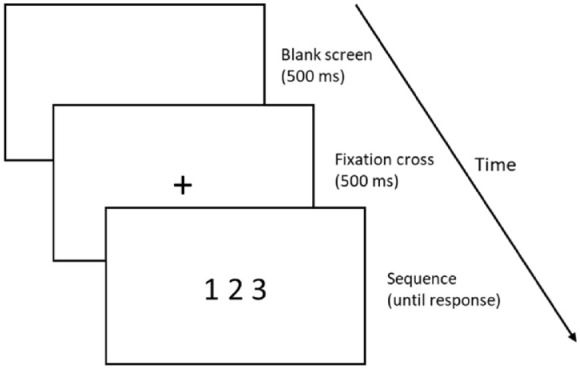
Timings of order judgement task.

A computerised order judgement task was created using PsychoPy (Peirce et al., 2019), and presented to participants via Pavlovia.org. This task was based on those used in similar studies (Vos et al., 2017). During the task, three-digit sequences (e.g., 1-2-3) were presented in the centre of the screen, and participants were asked to indicate whether these were “in order” or “not in order” using the “P” and “Q” keys on a QWERTY keyboard. “P” was used to indicate ordered sequences and “Q” was used to indicate non-ordered sequences. Sequences remained on screen until a key press was registered. This was followed by a blank screen, a fixation cross, and then the next sequence (exact timings shown in Figure 1). To ensure they understood the instructions, participants first completed 12 practice trials with on-screen feedback provided; although data from practice trials were not analysed. The practice trials included one of each unique sequence included in the task. No feedback was given during the critical trials.

Sequences were selected to ensure that participants had to process all three digits before being able to verify or reject a sequence. Furthermore, to reduce complexity, ordered sequences were ascending only, with a numerical distance between digits of either one (consecutive) or two (non-consecutive). Each ordered sequence (e.g., 1-2-3) had a corresponding non-ordered sequence (e.g., 2-3-1). This resulted in a total of 12 unique sequences (6 ordered, 6 non-ordered). For a full list of included sequences, see Table 1.

**Table 1.** All unique sequences included in the order judgement task.

Left	Centre	Right	Type	Distance
1	2	3	Ordered	1
2	3	1	Non-ordered	1
2	3	4	Ordered	1
3	4	2	Non-ordered	1
3	4	5	Ordered	1
4	5	3	Non-ordered	1
1	3	5	Ordered	2
3	5	1	Non-ordered	2
2	4	6	Ordered	2
4	6	2	Non-ordered	2
3	5	7	Ordered	2
5	7	3	Non-ordered	2

In both the mixed and blocked conditions, each unique sequence was presented four times, resulting in a total of 48 trials in each condition, and 96 trials in the full task. In the mixed condition, both consecutive and non-consecutive sequences were presented in an intermingled manner. In the blocked condition, consecutive and non-consecutive sequences were presented in separate blocks. The consecutive-only block contained both ordered consecutive sequences (e.g., 2-3-4) and their corresponding non-ordered consecutive sequences (e.g., 3-4-2). The non-consecutive-only block contained both ordered non-consecutive sequences (e.g., 2-4-6) and their corresponding non-ordered non-consecutive sequences (e.g., 4-6-2).

The order in which the two blocks were presented was counterbalanced. Furthermore, in the blocked condition, a short break needed to be included between the two blocks. Therefore, a break was also included at the same time point in the mixed condition. The order in which the mixed and blocked conditions were presented was also counterbalanced across participants. Therefore, there was a total of four possible presentation orders: CNMM, NCMM, MMCN, and MMNC (C = consecutive; N = non-consecutive; M = mixed). At the start of the task, participants were instructed that “in order” could refer to both consecutive and non-consecutive sequences. They were not informed that some blocks would include only consecutive or only non-consecutive sequences. Including breaks, the full task took approximately 6 min per participant.

### Results

Analyses for both experiments were conducted using the statistical programming language R within RStudio (R Core Team, 2020; RStudio Team, 2022). All data and analyses for this study can be found at osf.io/8rzm6. Data from three participants were removed due to one not completing the full task, one having both low overall accuracy (below 80%) and many individual response times below 200 ms, and another for having both low general accuracy (below 80%) and approximately chance accuracy on non-consecutive sequences. As such, data from 97 participants were included in the analysis. To determine the minimum effect size we could detect with a sample of 97 participants, we conducted a post hoc sensitivity analysis using G*Power 3.1 (Faul et al., 2009). This suggested that with an alpha of .05, power set to .95, and a known average correlation of 0.80 among repeated measures, a 2 × 2 repeated measures analysis of variance (ANOVA) would be able to detect a small minimum effect size of f = 0.095 or $\eta_{p}^{2}$ = .01.

Consistent with previous studies (Dubinkina et al., 2021), we removed responses that were shorter than 200 ms (*n* = 4; 0.04%) or longer than the overall mean plus three standard deviations (*M* = 1,083 ms, *SD* = 896 ms; *n* = 89; 0.96%) as these were considered unlikely to be genuine responses. As we were only interested in correct responses for correctly ordered sequences, we also removed incorrect responses (*n* = 249; 2.7%) and responses for incorrectly ordered sequences (*n* = 4,505; 50.22%). Although we did not analyse any accuracy effects, we note that mean accuracy (~97%) was approximately at ceiling across all participants.

Following this trimming procedure, the distribution of response times still appeared somewhat positively skewed (skewness = 1.96) and leptokurtic (kurtosis = 10.79). Therefore, to account for this, we considered only median response times for the remainder of the analysis (Whelan, 2008). We calculated median response times for each participant for each of the distance (consecutive/non-consecutive) and blocking (blocked/mixed) conditions. Means and standard deviations for each condition are shown in Table 2.

**Table 2.** Means and standard deviations for all condition/distance combinations.

Condition	Distance	Mean response time (ms)	Standard deviation
Blocked	Consecutive	869	323
	Non-consecutive	984	408
Mixed	Consecutive	866	276
	Non-consecutive	974	364

First, to evaluate the influence of condition and distance on response times, we conducted a 2 (condition: blocked, mixed) × 2 (distance: consecutive, non-consecutive) repeated measures ANOVA. In addition, we calculated Bayes factors using the BayesFactor package in R (Morey et al., 2022). We first calculated the BF_10_ values for the two main effects models separately, then together, and then also including the interaction between main effects. In all the models, subject was included as a random variable. Crucially, we compared the model including both main effects and the interaction against the model including only the two main effects by dividing the respective BF_10_ values. Bayes factor values greater than 1 indicate support for the alternative model, with values larger than 3 denoting at least moderate support. Conversely, values less than 1 indicate support for the null model, with values smaller than 0.33 indicating at least moderate evidence (Jeffreys, 1961).

**Figure 2.**
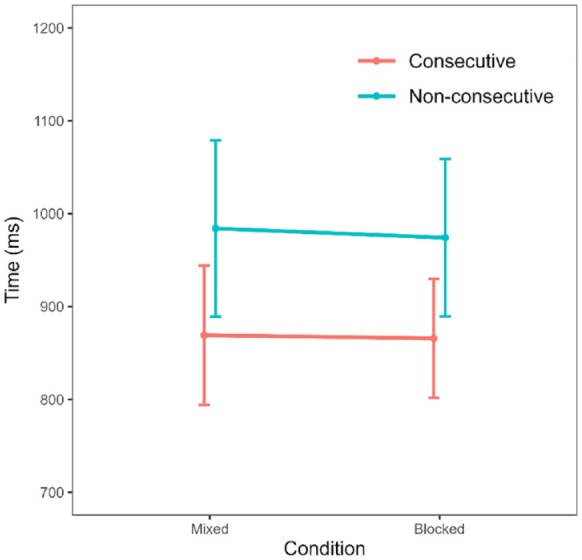
Median response times by condition. Error bars denote 95% confidence intervals.

The ANOVA indicated a significant main effect of distance, with consecutive sequences being processed faster than non-consecutive sequences, *F*(1, 96) = 33.65, *p* < .001, $\eta_{p}^{2}$ = .26. The BF_10_ value here (1.60 × 10^8^) suggests extreme evidence in favour of an effect of distance. However, there was no main effect of condition, *F*(1, 96) = 0.14, *p* = .710. In fact, the BF_10_ value (0.12) indicates moderate evidence against an effect of condition, suggesting there was no difference in response times between the mixed and blocked conditions. Furthermore, there was also no interaction between distance and condition, *F*(1, 96) = 0.09, *p* = .768. Accordingly, comparing the model including both main effects and the interaction against the model including only the two main effects returned a BF value of 0.13, suggesting moderate evidence against an interaction. These results are illustrated in Figure 2.

We also assessed the influence of presentation order on these findings, which suggested responses were faster in whichever condition participants completed second, regardless of which order the conditions were presented in (see the online Supplementary Material).

### Interim discussion

In Experiment 1, we tested whether presenting consecutive and non-consecutive sequences in separate blocks improved order processing performance, particularly on non-consecutive sequences. We argued that such improvement would be expected if participants found switching between concepts of order to be cognitively demanding. However, we instead found that response times did not vary depending on whether consequence and non-consecutive sequences were presented separately or within the same block. Therefore, at face value, these results suggest that switching between concepts of order during an order judgement task is not a cognitively demanding process, and does not drive the difference in response times between consecutive and non-consecutive sequences, at least not in adults. Beyond this, we observed a reverse distance effect in both the mixed and blocked conditions, as well as a practice effect whereby performance improved in whichever condition participants completed second.

A notable limitation of this experiment, however, is that our order judgement task was relatively short. For instance, only 24 trials were included in each of the consecutive-only and non-consecutive-only blocks. Therefore, from a cognitive demand perspective, it remains possible that adults do find it cognitively demanding to switch between consecutive and non-consecutive concepts of order, but our task was perhaps too short for this demand to be alleviated during the blocked condition. Therefore, to be able to determine whether switching between concepts of order is indeed a cognitively demanding process, it is necessary to first attempt to replicate the present findings using a longer version of the order judgement task.

Another important implication of these results concerns the count-list explanation of the reverse distance effect. More specifically, Gattas et al. (2021) proposed that when judging the order of sequences, adults likely implement a two-step process whereby they first check whether a sequence directly matches the count-list. Arguably, from this two-step perspective, presenting non-consecutive sequences separately should eliminate the need to first check whether a sequence matches the count-list. Therefore, we would have expected non-consecutive sequences to be processed faster in the blocked condition. In contrast, however, we observed similar response times for non-consecutive sequences in the mixed and blocked conditions.

A possible explanation for why we did not observe faster response times for non-consecutive sequences in the blocked condition may be that participants may simply not have realised when a block contained only consecutive or only non-consecutive sequences. Accordingly, from a two-step perspective, participants would still have been expected to process non-consecutive sequences by first checking whether they matched the count-list. Supporting this explanation, participants were not informed in advance when a block contained only consecutive or only non-consecutive sequences. In fact, they were not informed at all that the types of sequences presented would vary between blocks. Therefore, it is certainly plausible that participants might not have noticed when only non-consecutive sequences were presented. Because of this, the present findings are not sufficient to determine whether adults implement the two-step approach during order processing. A potential method to determine this, however, would be to modify the order judgement task to make the distinction between mixed and blocked conditions clearer to participants.

In Experiment 2, therefore, we modified our order judgement task to better test both the cognitive demands and two-step hypotheses. First, we doubled the number of trials to increase statistical power and allow more time for any cognitive demand effects to become noticeable. Second, we made the distinction between mixed and blocked conditions clearer to participants. This involved informing participants at the start of the task that sequence types would vary between blocks, as well as adding instructions before each block to inform participants which sequence types were about to be presented.

## Experiment 2

### Method

#### Participants

Another 100 UK-based participants (*M*_age_ = 39.80 years, *SD* = 14.35) were recruited via Prolific.co. Each participant received £2.50 for participating.

#### Order judgement task

For this experiment, we made three changes to the order judgement task used in Experiment 1, but all other aspects of the task remain unchanged (see Experiment 1 Method for details). First, we doubled the number of trials in each condition so that each unique sequence was now presented eight times, resulting in a total of 96 trials in both the mixed and blocked conditions, and 192 trials in the full task. Second, instructions given at the start of the task were modified to clarify that sequence types presented would vary between blocks of trials. Third, before each block, instructions were presented on screen to inform participants whether the following block would include consecutive only, non-consecutive only, or both consecutive and non-consecutive sequences. Including breaks, the full task took approximately 10 min per participant.

### Results

Data from four participants were removed due to their patterns of responses suggesting they may not have fully understood the task instructions. This included three participants with both low overall accuracy (below 80%) and below or at chance accuracy on non-consecutive sequences, and one participant with only slightly above chance accuracy on ordered non-consecutive sequences. Consequently, data from 96 participants were included in the analysis. To determine the minimum effect size we could detect with a sample of 96 participants, we conducted a post hoc sensitivity analysis using G*Power 3.1 (Faul et al., 2009). This suggested that with an alpha of .05, power set to .95, and a known average correlation of 0.77 among repeated measures, a 2 × 2 repeated measures ANOVA would be able to detect a small minimum effect size of f = 0.102 or $\eta_{p}^{2}$ = .01.

As in the first experiment, we removed responses that were shorter than 200 ms (*n* = 4; 0.02%) or longer than the overall mean plus three standard deviations (*M* = 956 ms, *SD* = 872 ms; *n* = 98; 0.53%) as these were considered unlikely to be genuine responses. Because we are only interested in correct responses for correctly ordered sequences, we also removed incorrect responses (*n* = 418; 2.28%) and responses for incorrectly ordered sequences (*n* = 8955; 49.99%). As in Experiment 1, we did not analyse any accuracy effects, but we again observed mean accuracy (~98%) to be approximately at ceiling across all participants.

Following this trimming procedure, the distribution of response times still appeared somewhat positively skewed (skewness = 1.93) and leptokurtic (kurtosis = 11.41). Therefore, to account for this, we again considered only median response times for the remainder of the analysis. We calculated median response times for each participant for each of the distance (consecutive/non-consecutive) and blocking (blocked/mixed) conditions. Means and standard deviations for each condition are shown in Table 3.

**Table 3.** Means and standard deviations for all condition/distance combinations.

Condition	Distance	Mean response time (ms)	Standard deviation
Blocked	Consecutive	754	231
	Non-consecutive	860	319
Mixed	Consecutive	763	221
	Non-consecutive	851	267

**Figure 3.**
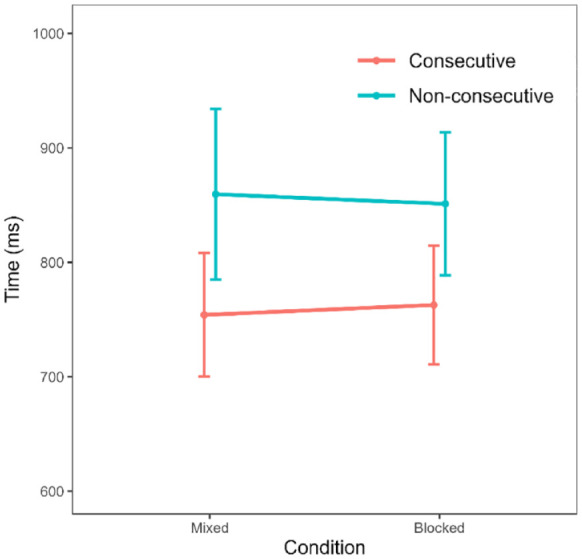
Median response times by condition. Error bars denote 95% confidence intervals.

To evaluate the influence of condition and distance on response times, we conducted a 2 (condition: blocked, mixed) × 2 (distance: consecutive, non-consecutive) repeated measures ANOVA. This revealed a significant effect of distance, with consecutive sequences being processed faster than non-consecutive sequences, *F*(1, 95) = 44.60, *p* < .001, $\eta_{p}^{2}$ = .32. The BF_10_ value here (1.54 × 10^9^) suggests extreme evidence in favour of an effect of distance. However, there was no main effect of condition, *F*(1, 95) = 0.00, *p* = .995. In fact, as in Experiment 1, the BF_10_ value (0.11) indicates moderate evidence against an effect of condition, suggesting there was no difference in response times between the mixed and blocked conditions. Furthermore, there was also no interaction between distance and condition, *F*(1, 95) = 0.88, *p* = .350. Accordingly, comparing the model including both main effects and the interaction against the model including only the two main effects returned a BF value of 0.16, suggesting moderate evidence against an interaction. These results are visualised in Figure 3.

As in Experiment 1, we also assessed the influence of presentation order on these findings, which again suggested responses were faster in whichever condition participants completed second, regardless of which order the conditions were presented in (see the online Supplementary Material).

## General discussion

In this study, we investigated the impact of switching between consecutive and non-consecutive concepts of order on order processing performance. More specifically, we tested whether presenting consecutive and non-consecutive sequences separately would eliminate the need to switch between concepts of order and thus would also improve order processing speed, in particular, for non-consecutive sequences. In contrast to our expectations, however, we observed similar patterns of response times independent of whether sequences were presented separately or together. Moreover, this finding remained even when we increased the number of trials and repeatedly emphasised the distinction between mixed and blocked conditions to participants. Consequently, our findings suggest that adults do not find it cognitively demanding to switch between consecutive and non-consecutive concepts of order. This suggests, therefore, that adults’ slower processing of non-consecutive sequences cannot be attributed to an increased cognitive demand. Beyond this, participants in both experiments displayed reverse distance effects in both the mixed and blocked conditions, as well as practice effects whereby performance improved in whichever condition they completed second.

In addition to suggesting adults do not find it cognitively demanding to switch between concepts of order, the present findings also suggest adults likely do not implement a two-step approach during order judgement tasks. From the two-step perspective, non-consecutive sequences are processed slower because judging their order requires multiple steps, starting with first checking whether the sequence matches the count-list (Gattas et al., 2021). Therefore, by presenting consecutive and non-consecutive sequences separately, our blocking condition should have eliminated the need to undertake this first step and thus should have improved response times. Despite this, however, we observed similar patterns of responses between the mixed and blocked conditions in both experiments, suggesting that slower response times for non-consecutive sequences likely do not result from adults implementing such a two-step process when judging the ordinality of non-consecutive sequences. It should be acknowledged, however, that it remains possible such a two-step process is implemented during order processing, but that its implementation is simply more automatic than strategic. Accordingly, the present manipulation is unlikely to have influenced such an automatic process.

Nonetheless, this general lack of support for the two-step perspective arguably supports the facilitation account of the reverse distance effect. This is because there are currently two main ways of interpreting the reverse distance effect; either this effect results from consecutive sequences being facilitated or from non-consecutive sequences being impeded. Therefore, the lack of evidence suggesting non-consecutive sequences are impeded by a two-step process is consistent with the interpretation that the reverse distance effect results from consecutive sequences being facilitated. More specifically, it is thought that more familiar sequences (e.g., 1-2-3) are processed faster due to being more easily retrieved from long-term memory (Sella et al., 2020). Therefore, because consecutive sequences are more familiar than non-consecutive sequences, their processing is more likely to be facilitated by memory-retrieval processes (Devlin et al., 2022; Vos et al., 2017, 2021). Nonetheless, this facilitation account of the reverse distance effect in adults provides no obvious explanation for why many young children display consistently low accuracy when judging the ordinality of non-consecutive sequences (Gilmore & Batchelor, 2021; Hutchison et al., 2022).

When considering children’s low accuracy on non-consecutive sequences, we proposed that the order judgement task may simply be too cognitively demanding for some children due to the need to repeatedly switch between concepts of order. In a sample of adults, we assessed whether eliminating this need would improve performance. However, because we observed no such improvement, we found no support for the notion that switching between concepts of order is cognitively demanding. It is possible this switching is only noticeably demanding for individuals with low executive functioning skills; therefore, any future replications of this design should consider including executive functioning measures. Furthermore, the lack of an effect in adults does not rule out that switching between concepts of order is cognitively demanding for children. Therefore, we suggest replicating the present design in children as a priority for future research. This could involve testing not just whether presenting consecutive and non-consecutive sequences separately improves performance, but also whether any such improvement occurs disproportionately for those children who typically reject non-consecutive sequences. This would help establish not just whether children find it cognitively demanding to switch between concepts of order, but also whether this demand explains the tendency to reject non-consecutive sequences.

Nonetheless, the lack of empirical support for the cognitive demand explanation highlights an important unresolved inconsistency in the literature. In particular, the fact that children considered to lack conceptual understanding that non-consecutive sequences are correctly ordered (Hutchison et al., 2022) still appear able to correctly produce ordered non-consecutive sequences during order production tasks (Bugden & Brannon, 2021). Because we failed to reconcile these findings from a cognitive demand perspective, alternative possibilities should be considered. One such possibility is that order production tasks do not actually require a comprehensive understanding of ordinality. Instead, children lacking this understanding may produce ordered non-consecutive sequences by implementing some kind of strategy such as identifying the smallest number and then counting upwards. Regardless, understanding why performance differs between order production and order verification tasks has important theoretical implications for our understanding of how children’s understanding of ordinality develops. Therefore, we encourage further investigating the mechanisms and strategies underlying order production tasks as a key direction for future research.

One potential limitation of this study is that data were collected online; thus, one might suspect potential issues with data quality preventing observation of small differences in response time. For instance, participant inattentiveness has been suggested as potential concern with online data collection (Newman et al., 2021). However, we first wish to highlight that we collected data using Prolific, which has been shown to produce higher quality data than platforms such as MTurk (Peer et al., 2022). Furthermore, we note that participants displayed both near ceiling accuracy and clear reverse distance effects in both experiments. Therefore, not only does this provide confidence that participants fully understood the task instructions, but it also demonstrates that it was possible to detect relatively small differences in response times. This is consistent with findings that other numerical cognition effects, such as the standard distance effect, have also been reliably replicated using online methods (Kochari, 2019). Accordingly, we are confident that the lack of difference observed between the mixed and blocked conditions is a genuine property of the sample, rather than a limitation of online data collection.

Another point for consideration concerns the difference in mean response times between the two experiments. Notably, participants in Experiment 2 were approximately 100 ms faster on average compared to participants in Experiment 1. In our view, the most probable explanation for this finding is that it reflects a practice effect. For instance, Experiment 2 included twice as many trials as Experiment 1. Consequently, participants trained for longer on the task in Experiment 2, which most likely resulted in faster response times overall. This rationale is backed by the finding that participants in both experiments exhibited practice effects whereby their responses were significantly faster in the second half of the experiment compared to the first. In fact, when comparing response times from the full task in Experiment 1 to just the first half of the task in Experiment 2, the difference in response times between the two experiments was no longer statistically significant (see the Online Supplementary Material). Accordingly, the faster response times in Experiment 2 most likely resulted from the greater number of trials allowing more time for practice effects to occur.

In conclusion, we found no evidence to substantiate our claim that switching between consecutive and non-consecutive concepts of order during order judgement tasks underlies the observed disadvantage in processing non-consecutive sequences. Furthermore, the lack of any such cognitive demand effects in adults suggests the reverse distance effect in adults likely does not result from the implementation of a two-step process when judging non-consecutive sequences. Importantly, however, the absence of a cognitive demand effect in adults does not necessarily mean that such an effect is not present in children. Therefore, we suggest replicating the present design in children as a priority for future research. Finally, in absence of support for the cognitive demand explanation, we emphasise the need to consider alternative explanations for why some children appear to consider non-consecutive sequences as incorrectly ordered during order judgement tasks but correctly ordered during order production tasks.

## Supplemental Material

sj-docx-1-qjp-10.1177_17470218231220912 – Supplemental material for Concepts of order: Why is ordinality processed slower and less accurately for non-consecutive sequences? Supplemental material, sj-docx-1-qjp-10.1177_17470218231220912 for Concepts of order: Why is ordinality processed slower and less accurately for non-consecutive sequences? by Declan Devlin, Korbinian Moeller, Iro Xenidou-Dervou, Bert Reynvoet and Francesco Sella in Quarterly Journal of Experimental Psychology
