# Supplementary material for: Concepts of order: Why is ordinality processed slower and less accurately for non-consecutive sequences?
Source: Q J Exp Psychol (Hove). 2024 Jan 11;77(8):1610–9. doi: 10.1177/17470218231220912 (PMC11295408; doi:10.1177/17470218231220912)
Supplement: sj-docx-1-qjp-10.1177_17470218231220912 – Supplemental material for Concepts of order: Why is ordinality processed slower and less accurately for non-consecutive sequences? [file sj-docx-1-qjp-10.1177_17470218231220912.docx]

**Supplementary materials – presentation order effects**

**Experiment 1**

We also considered the impact of presentation order on these findings. Therefore, we conducted a mixed factorial ANOVA with one between-participants factor of order (mixed-then-blocked, blocked-then-mixed) and the same two within-participants factors of distance (consecutive, non-consecutive) and condition (blocked, mixed). This revealed no main effect of presentation order, *F*(1, 95) = 1.43, *p* = .235. However, there was a significant interaction between presentation order and condition, *F*(1, 95) = 39.89, *p* <.001, $\eta_{p}^{2}$ = .30. This interaction appeared to reflect a practice effect whereby performance improved in whichever condition participants completed second. Therefore, to test for this, we re-ran this ANOVA, but this time replacing condition with a variable representing whether the condition was completed in the first or second half of the experiment. This revealed a significant effect of half, with responses being faster in the second half of the task compared to the first, *F*(1, 95) = 39.89, *p* < .001, $\eta_{p}^{2}$ = .30. Importantly, however, there was no interaction between presentation order and half, suggesting responses were faster in whichever condition participants completed second, regardless of which order the conditions were presented in, *F*(1, 95) = 0.00, *p* = .988.

**Experiment 2**

Next, we again considered the impact of presentation order on these findings. Accordingly, we conducted a mixed factorial ANOVA with the between-participants factor of order (mixed-then-blocked, blocked-then-mixed) and the same two within-participants factors of distance (consecutive, non-consecutive) and condition (blocked, mixed). This revealed no main effect of presentation order, *F*(1, 94) = 1.89, *p* = .172. However, there was a significant interaction between presentation order and distance, *F*(1, 94) = 5.29, *p* = 0.24, $\eta_{p}^{2}$ = .053, suggesting there was a stronger reverse distance effect in the blocked-then-mixed order than in the mixed-then-blocked order. Furthermore, there was a significant interaction between presentation order and condition, *F*(1, 94) = 48.83, *p* <.001, $\eta_{p}^{2}$ = .34.

As in the first experiment, the interaction between presentation order and condition appeared to reflect a practice effect whereby performance improved in whichever condition participants completed second. Therefore, to test for this, we re-ran the above ANOVA, but this time replacing condition with a variable coding whether the respective condition was completed in the first or second half of the experiment. This revealed a significant effect of half, with responses being faster in the second half of the task compared to the first, *F*(1, 94) = 48.83, *p* < .001, $\eta_{p}^{2}$ = .34. Importantly, however, there was no interaction between presentation order and half, suggesting responses were faster in whichever condition participants completed second, regardless of which order the conditions were presented in, *F*(1, 94) = 0.02, *p* = .879.

**Quantifying practice effects**

A post-hoc independent samples *t*-test revealed responses were faster in the second experiment (mean = 807 ms, SD = 248) compared to the first experiment (mean = 923 ms, SD = 327), *t*(191) = 2.89, *p* = .004.

A possible explanation for this finding is that the faster response times in experiment 2 result from this experiment including twice as many trials as the task in experiment 1. As such, the longer duration of the task arguably allowed more time for participants to train on the task. Supporting this explanation, we observed practice effects in both experiments whereby responses were faster in the second half of the task compared to the first. For instance, in experiment 1, a post-hoc paired-samples *t*-test revealed responses were slower in the first half of the task (mean = 970 ms, SD = 355) compared to the second half (mean = 876 ms, SD = 291), *t*(96) = 6.36, *p* < .001. Similarly, in experiment 2, responses were slower in the first half of the task (mean = 852 ms, SD = 262) compared to the second half (mean = 762 ms, SD = 225), *t*(95) = 7.02, *p* < .001.

To account for the difference in task lengths, therefore, we compared response times from the full task in experiment 1 to just the first half of the task in experiment 2. Notably, the differences in responses times between the two experiments were now no longer significant, *t*(191) = 1.71, *p* = .089.
